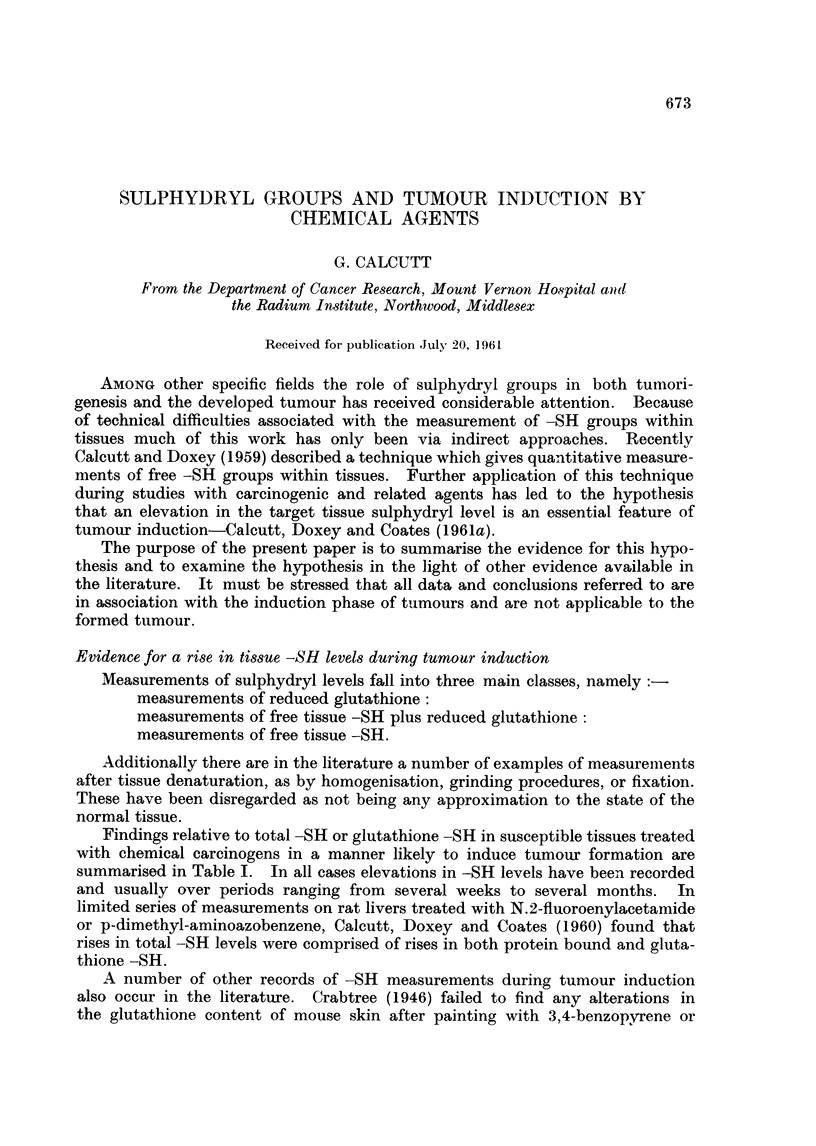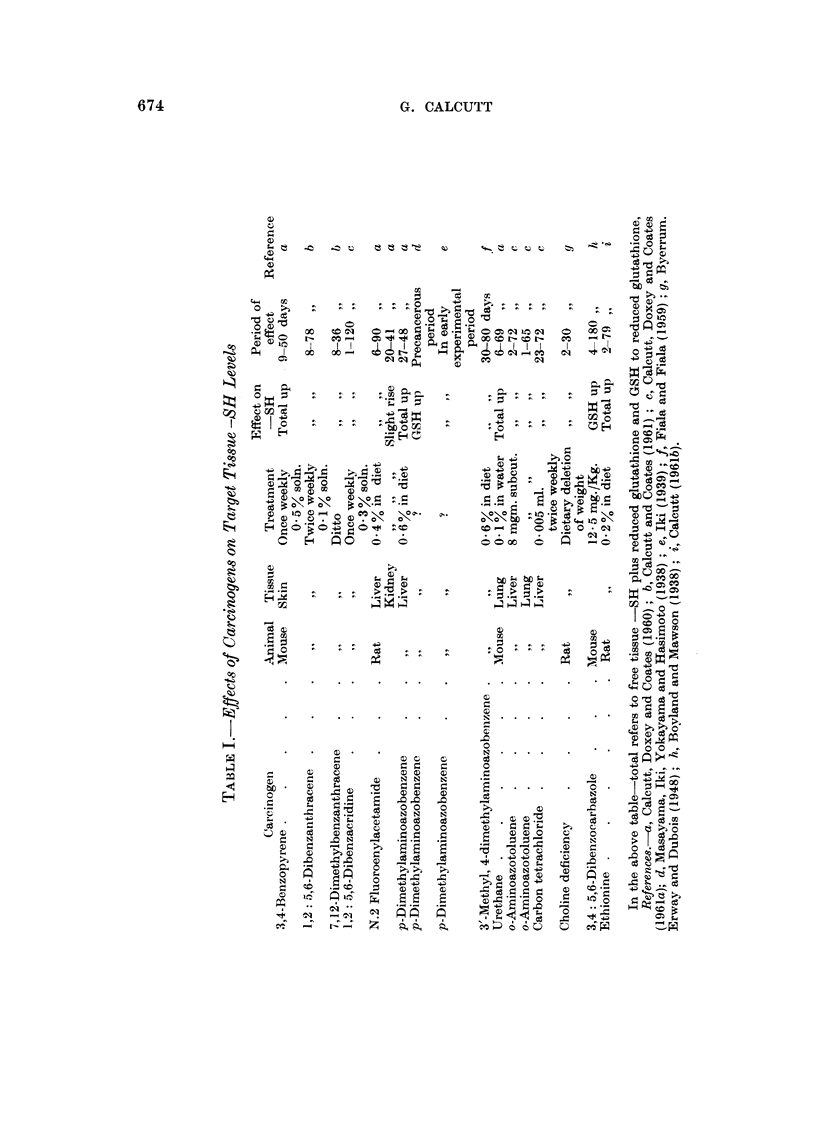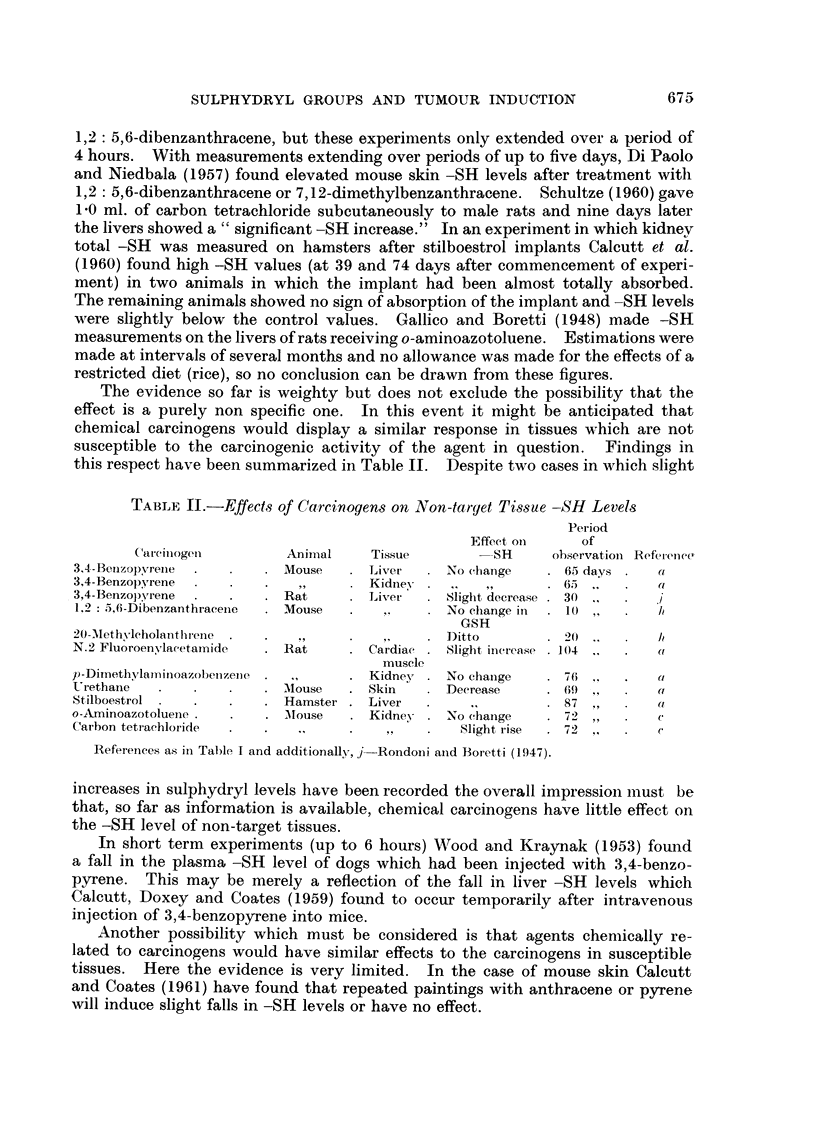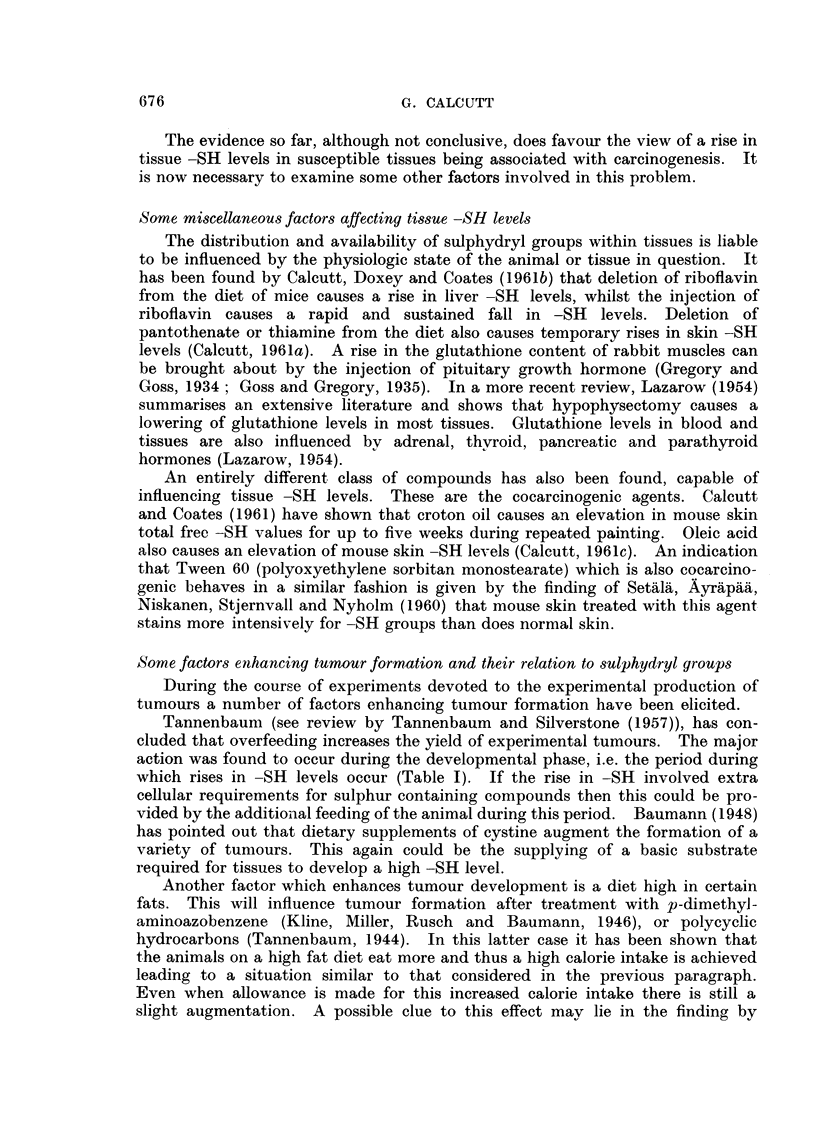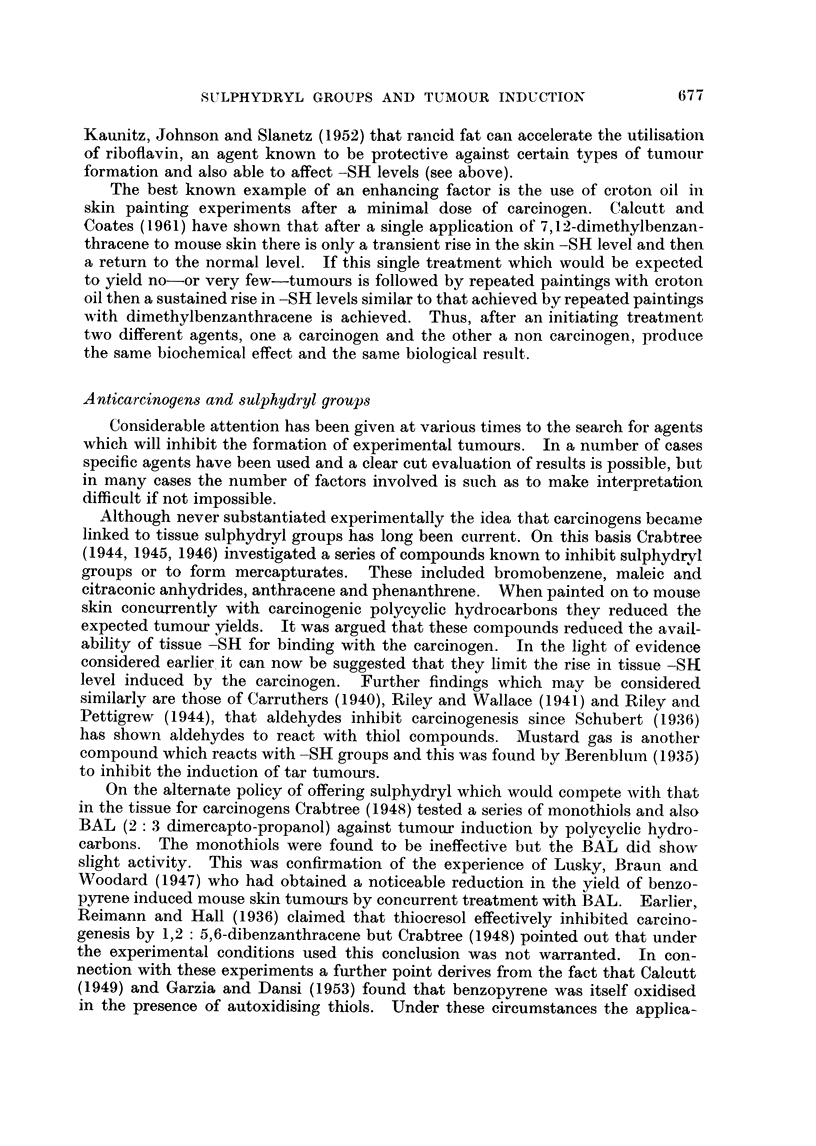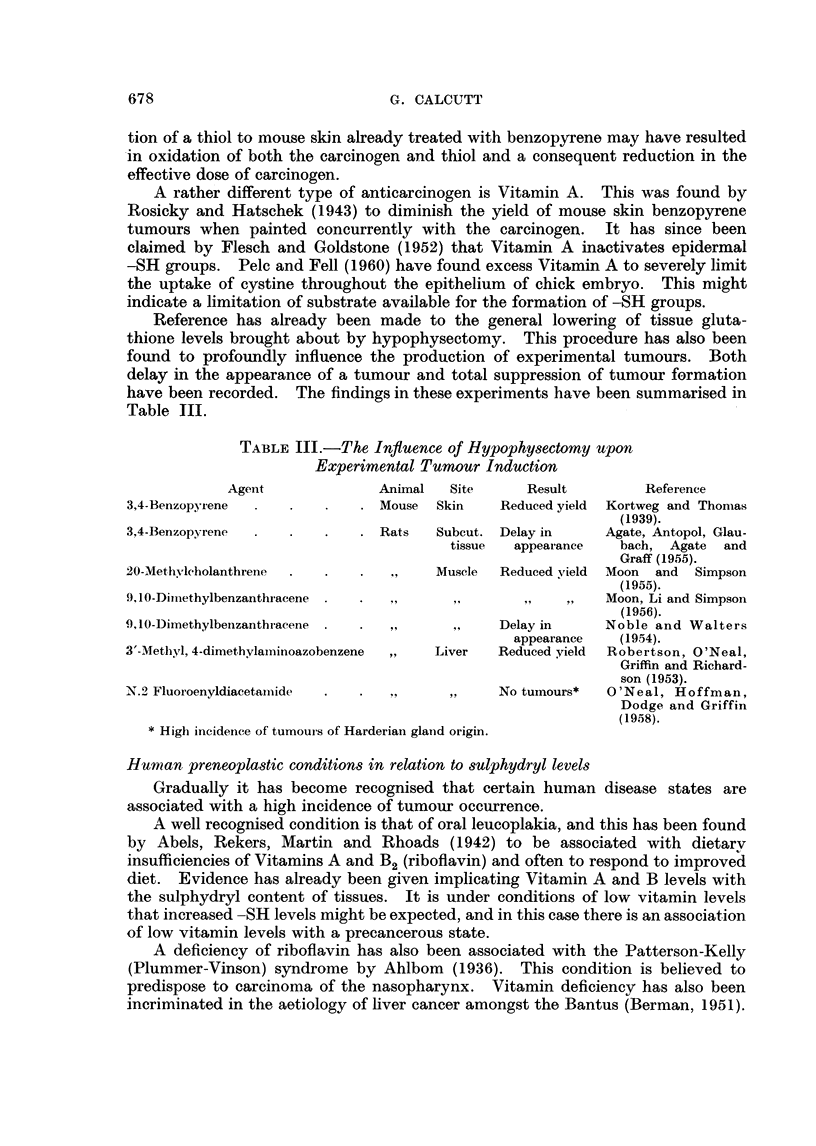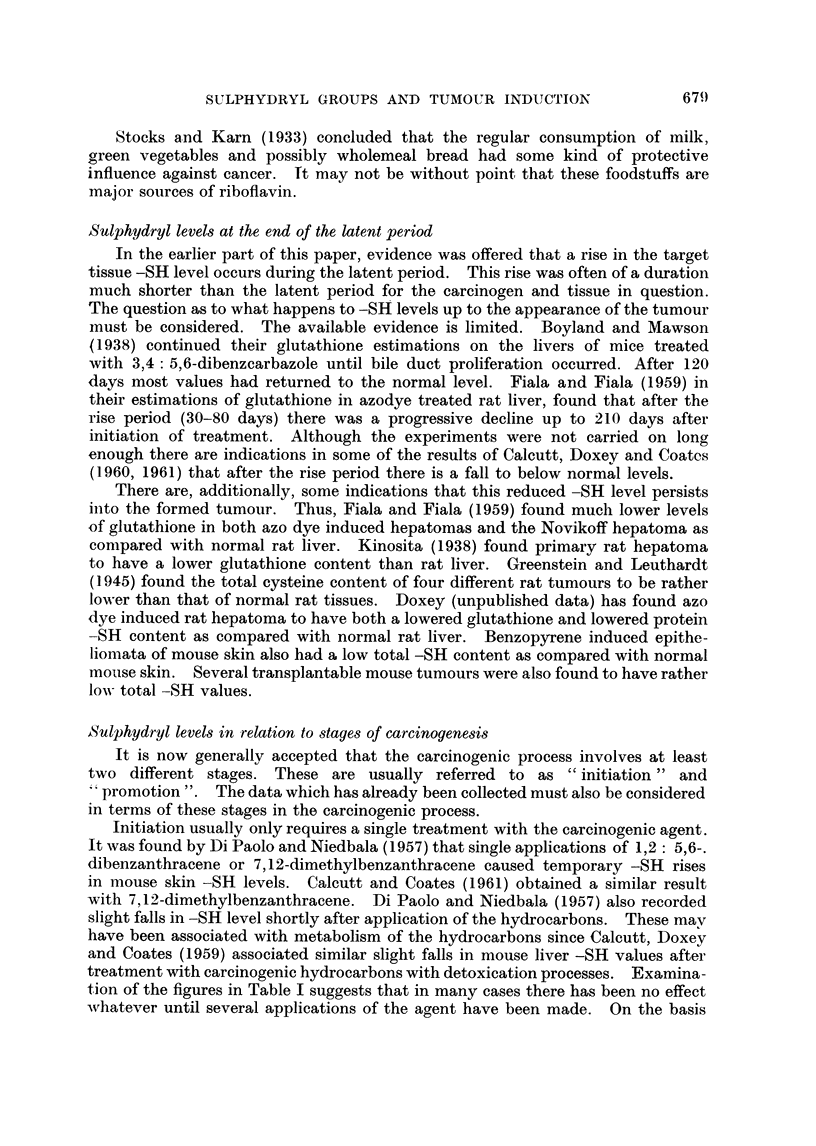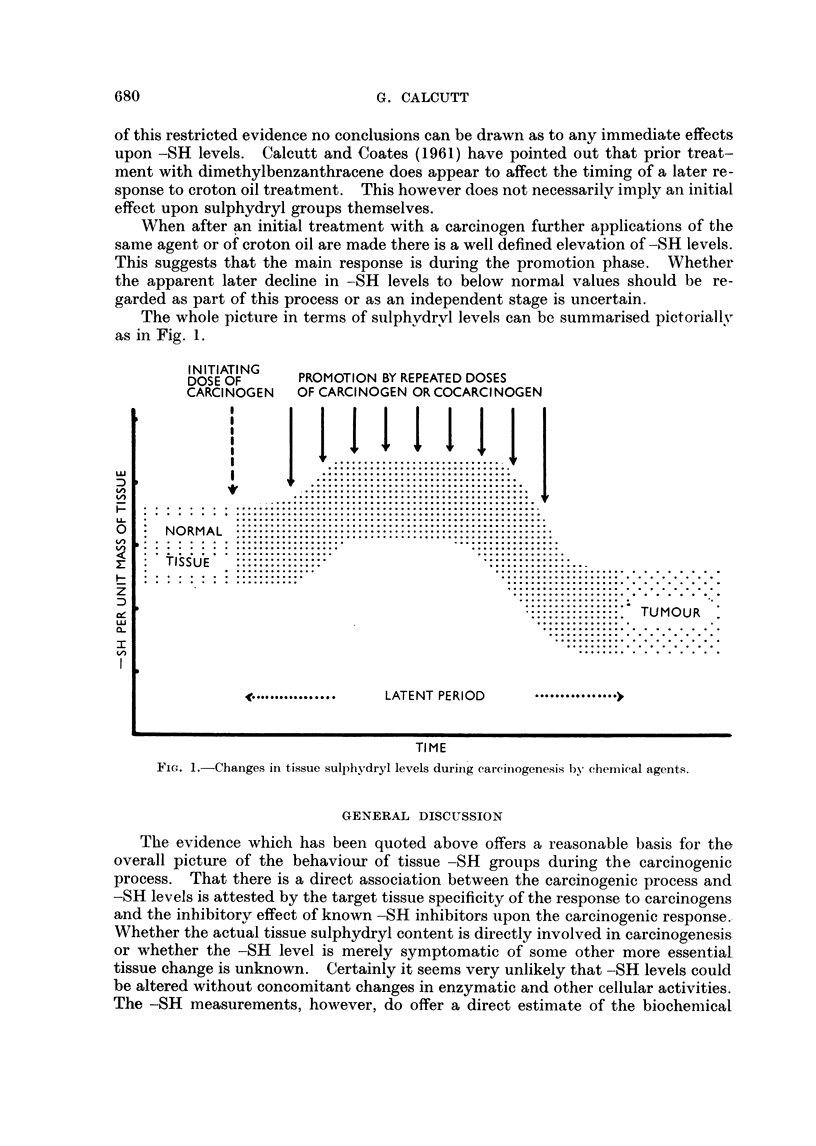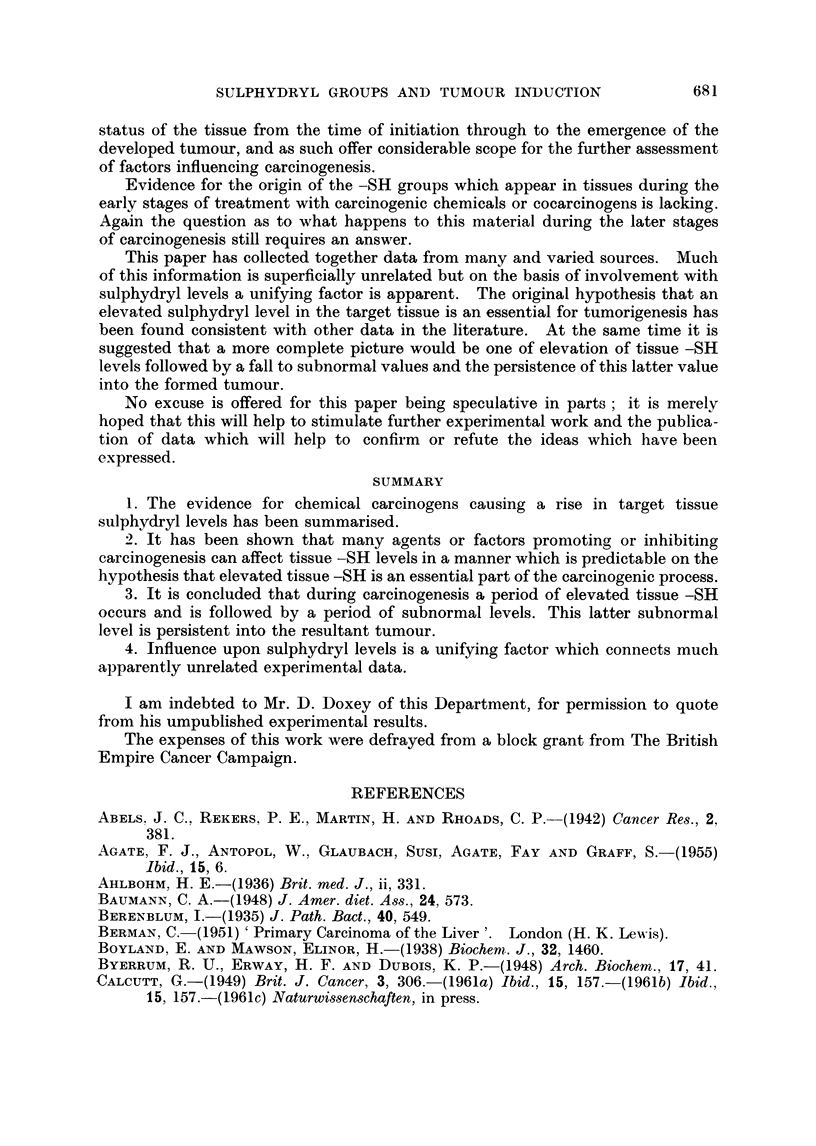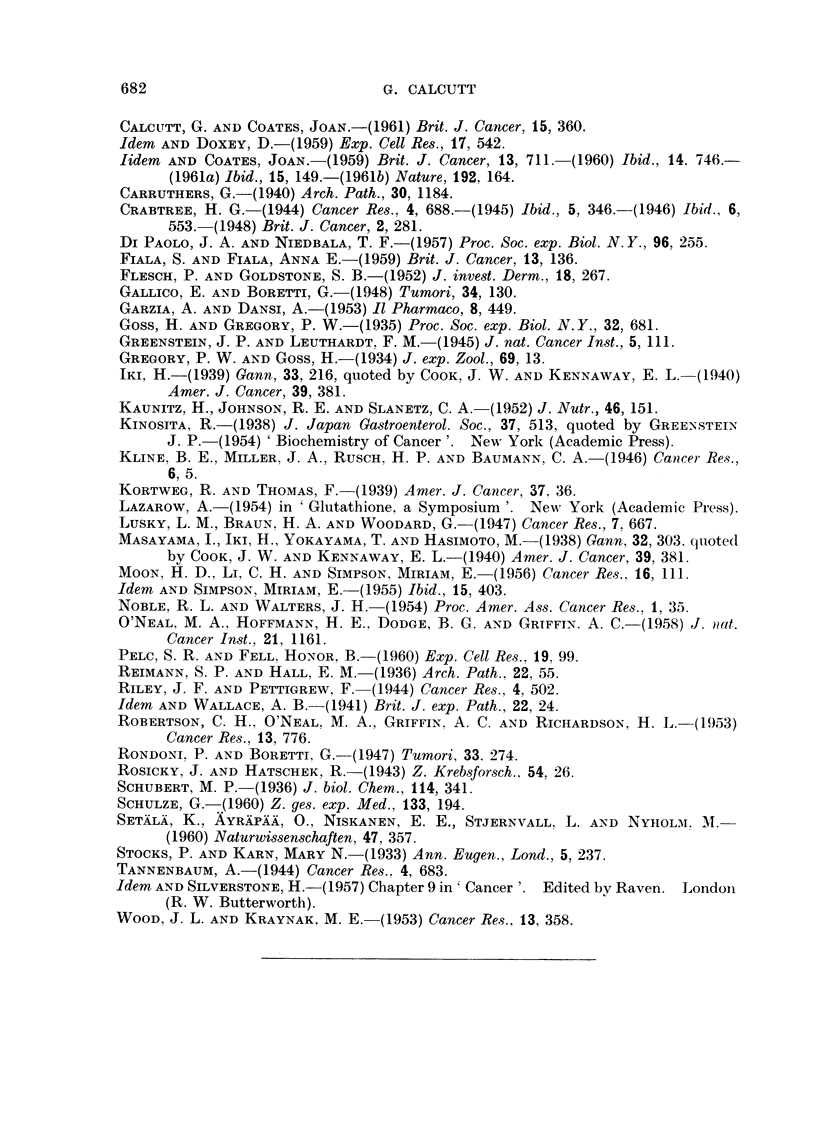# Sulphydryl Groups and Tumour Induction by Chemical Agents

**DOI:** 10.1038/bjc.1961.76

**Published:** 1961-09

**Authors:** G. Calcutt


### SULPHYDRYL GROUPS AND TUMOUR INDUCTION BY CHEMICAL AGENTS

G. CALCUTT

*From the Department of Cancer Research, Mount Vernon Hospital and the Radium Institute, Northwood, Middlesex*

Received for publication July 20, 1961

AMONG other specific fields the role of sulphydryl groups in both tumorigenesis and the developed tumour has received considerable attention. Because of technical difficulties associated with the measurement of –SH groups within tissues much of this work has only been via indirect approaches. Recently Calcutt and Doxey (1959) described a technique which gives quantitative measurements of free –SH groups within tissues. Further application of this technique during studies with carcinogenic and related agents has led to the hypothesis that an elevation in the target tissue sulphydryl level is an essential feature of tumour induction—Calcutt, Doxey and Coates (1961*a*).

The purpose of the present paper is to summarise the evidence for this hypothesis and to examine the hypothesis in the light of other evidence available in the literature. It must be stressed that all data and conclusions referred to are in association with the induction phase of tumours and are not applicable to the formed tumour.

*Evidence for a rise in tissue –SH levels during tumour induction*

Measurements of sulphydryl levels fall into three main classes, namely :—

measurements of reduced glutathione :
measurements of free tissue –SH plus reduced glutathione :
measurements of free tissue –SH.

Additionally there are in the literature a number of examples of measurements after tissue denaturation, as by homogenisation, grinding procedures, or fixation. These have been disregarded as not being any approximation to the state of the normal tissue.

Findings relative to total –SH or glutathione –SH in susceptible tissues treated with chemical carcinogens in a manner likely to induce tumour formation are summarised in Table I. In all cases elevations in –SH levels have been recorded and usually over periods ranging from several weeks to several months. In limited series of measurements on rat livers treated with N.2-fluoroenylacetamide or p-dimethyl-aminoazobenzene, Calcutt, Doxey and Coates (1960) found that rises in total –SH levels were comprised of rises in both protein bound and glutathione –SH.

A number of other records of –SH measurements during tumour induction also occur in the literature. Crabtree (1946) failed to find any alterations in the glutathione content of mouse skin after painting with 3,4-benzopyrene or

TABLE I.—*Effects of Carcinogens on Target Tissue -SH Levels*

| Carcinogen | Animal | Tissue | Treatment | Effect on —SH | Period of effect | Reference |
|---|---|---|---|---|---|---|
| 3,4-Benzopyrene . . . . . | Mouse | Skin | Once weekly 0·5% soln. | Total up | 9–50 days | *a* |
| 1,2 : 5,6-Dibenzanthracene . . . . | ,, | ,, | Twice weekly 0·1% soln. | ,, ,, | 8–78 ,, | *b* |
| 7,12-Dimethylbenzanthracene . . | ,, | ,, | Ditto | ,, ,, | 8–36 ,, | *b* |
| 1,2 : 5,6-Dibenzacridine . . . . | ,, | ,, | Once weekly 0·3% soln. | ,, ,, | 1–120 ,, | *c* |
| N.2 Fluoroenylacetamide . . . | Rat | Liver | 0·4% in diet | ,, ,, | 6–90 ,, | *a* |
| | | Kidney | ,, ,, ,, | Slight rise | 20–41 ,, | *a* |
| *p*-Dimethylaminoazobenzene . . | ,, | Liver | 0·6% in diet | Total up | 27–48 ,, | *a* |
| *p*-Dimethylaminoazobenzene . . | ,, | ,, | ? | GSH up | Precancerous period | *d* |
| *p*-Dimethylaminoazobenzene . . | ,, | ,, | ? | ,, ,, | In early experimental period | *e* |
| 3′-Methyl, 4-dimethylaminoazobenzene . | ,, | ,, | 0·6% in diet | ,, ,, | 30–80 days | *f* |
| Urethane . . . . . . . | Mouse | Lung | 0·1% in water | Total up | 6–69 ,, | *a* |
| *o*-Aminoazotoluene . . . . . | ,, | Liver | 8 mgm. subcut. | ,, ,, | 2–72 ,, | *c* |
| *o*-Aminoazotoluene . . . . . | ,, | Lung | ,, ,, | ,, ,, | 1–65 ,, | *c* |
| Carbon tetrachloride . . . . . | ,, | Liver | 0·005 ml. twice weekly | ,, ,, | 23–72 ,, | *c* |
| Choline deficiency . . . . . | Rat | ,, | Dietary deletion of weight | ,, ,, | 2–30 ,, | *g* |
| 3,4 : 5,6-Dibenzocarbazole . . . | Mouse | | 12·5 mg./Kg. | GSH up | 4–180 ,, | *h* |
| Ethionine . . . . . . . | Rat | ,, | 0·2% in diet | Total up | 2–79 ,, | *i* |

In the above table—total refers to free tissue —SH plus reduced glutathione and GSH to reduced glutathione,

*References.*—*a*, Calcutt, Doxey and Coates (1960) ; *b*, Calcutt and Coates (1961) ; *c*, Calcutt, Doxey and Coates (1961*a*); *d*, Masayama, Iki, Yokayama and Hasimoto (1938) ; *e*, Iki (1939) ; *f*, Fiala and Fiala (1959) ; *g*, Byerrum. Erway and Dubois (1948) ; *h*, Boyland and Mawson (1938) ; *i*, Calcutt (1961*b*).

1,2 : 5,6-dibenzanthracene, but these experiments only extended over a period of 4 hours. With measurements extending over periods of up to five days, Di Paolo and Niedbala (1957) found elevated mouse skin –SH levels after treatment with 1,2 : 5,6-dibenzanthracene or 7,12-dimethylbenzanthracene. Schultze (1960) gave 1·0 ml. of carbon tetrachloride subcutaneously to male rats and nine days later the livers showed a " significant –SH increase." In an experiment in which kidney total –SH was measured on hamsters after stilboestrol implants Calcutt *et al.* (1960) found high –SH values (at 39 and 74 days after commencement of experiment) in two animals in which the implant had been almost totally absorbed. The remaining animals showed no sign of absorption of the implant and –SH levels were slightly below the control values. Gallico and Boretti (1948) made –SH measurements on the livers of rats receiving *o*-aminoazotoluene. Estimations were made at intervals of several months and no allowance was made for the effects of a restricted diet (rice), so no conclusion can be drawn from these figures.

The evidence so far is weighty but does not exclude the possibility that the effect is a purely non specific one. In this event it might be anticipated that chemical carcinogens would display a similar response in tissues which are not susceptible to the carcinogenic activity of the agent in question. Findings in this respect have been summarized in Table II. Despite two cases in which slight

TABLE II.—*Effects of Carcinogens on Non-target Tissue –SH Levels*

| Carcinogen | Animal | Tissue | Effect on —SH | Period of observation | Reference |
|---|---|---|---|---|---|
| 3,4-Benzopyrene | Mouse | Liver | No change | 65 days | *a* |
| 3,4-Benzopyrene | ,, | Kidney | ,, ,, | 65 ,, | *a* |
| 3,4-Benzopyrene | Rat | Liver | Slight decrease | 30 ,, | *j* |
| 1,2 : 5,6-Dibenzanthracene | Mouse | ,, | No change in GSH | 10 ,, | *h* |
| 20-Methylcholanthrene | ,, | ,, | Ditto | 20 ,, | *h* |
| N.2 Fluoroenylacetamide | Rat | Cardiac muscle | Slight increase | 104 ,, | *a* |
| *p*-Dimethylaminoazobenzene | ,, | Kidney | No change | 76 ,, | *a* |
| Urethane | Mouse | Skin | Decrease | 69 ,, | *a* |
| Stilboestrol | Hamster | Liver | ,, | 87 ,, | *a* |
| *o*-Aminoazotoluene | Mouse | Kidney | No change | 72 ,, | *c* |
| Carbon tetrachloride | ,, | ,, | Slight rise | 72 ,, | *c* |

References as in Table I and additionally, *j*—Rondoni and Boretti (1947).

increases in sulphydryl levels have been recorded the overall impression must be that, so far as information is available, chemical carcinogens have little effect on the –SH level of non-target tissues.

In short term experiments (up to 6 hours) Wood and Kraynak (1953) found a fall in the plasma –SH level of dogs which had been injected with 3,4-benzopyrene. This may be merely a reflection of the fall in liver –SH levels which Calcutt, Doxey and Coates (1959) found to occur temporarily after intravenous injection of 3,4-benzopyrene into mice.

Another possibility which must be considered is that agents chemically related to carcinogens would have similar effects to the carcinogens in susceptible tissues. Here the evidence is very limited. In the case of mouse skin Calcutt and Coates (1961) have found that repeated paintings with anthracene or pyrene will induce slight falls in –SH levels or have no effect.

The evidence so far, although not conclusive, does favour the view of a rise in tissue –SH levels in susceptible tissues being associated with carcinogenesis. It is now necessary to examine some other factors involved in this problem.

*Some miscellaneous factors affecting tissue –SH levels*

The distribution and availability of sulphydryl groups within tissues is liable to be influenced by the physiologic state of the animal or tissue in question. It has been found by Calcutt, Doxey and Coates (1961*b*) that deletion of riboflavin from the diet of mice causes a rise in liver –SH levels, whilst the injection of riboflavin causes a rapid and sustained fall in –SH levels. Deletion of pantothenate or thiamine from the diet also causes temporary rises in skin –SH levels (Calcutt, 1961*a*). A rise in the glutathione content of rabbit muscles can be brought about by the injection of pituitary growth hormone (Gregory and Goss, 1934 ; Goss and Gregory, 1935). In a more recent review, Lazarow (1954) summarises an extensive literature and shows that hypophysectomy causes a lowering of glutathione levels in most tissues. Glutathione levels in blood and tissues are also influenced by adrenal, thyroid, pancreatic and parathyroid hormones (Lazarow, 1954).

An entirely different class of compounds has also been found, capable of influencing tissue –SH levels. These are the cocarcinogenic agents. Calcutt and Coates (1961) have shown that croton oil causes an elevation in mouse skin total free –SH values for up to five weeks during repeated painting. Oleic acid also causes an elevation of mouse skin –SH levels (Calcutt, 1961*c*). An indication that Tween 60 (polyoxyethylene sorbitan monostearate) which is also cocarcinogenic behaves in a similar fashion is given by the finding of Setälä, Äyräpää, Niskanen, Stjernvall and Nyholm (1960) that mouse skin treated with this agent stains more intensively for –SH groups than does normal skin.

*Some factors enhancing tumour formation and their relation to sulphydryl groups*

During the course of experiments devoted to the experimental production of tumours a number of factors enhancing tumour formation have been elicited.

Tannenbaum (see review by Tannenbaum and Silverstone (1957)), has concluded that overfeeding increases the yield of experimental tumours. The major action was found to occur during the developmental phase, i.e. the period during which rises in –SH levels occur (Table I). If the rise in –SH involved extra cellular requirements for sulphur containing compounds then this could be provided by the additional feeding of the animal during this period. Baumann (1948) has pointed out that dietary supplements of cystine augment the formation of a variety of tumours. This again could be the supplying of a basic substrate required for tissues to develop a high –SH level.

Another factor which enhances tumour development is a diet high in certain fats. This will influence tumour formation after treatment with *p*-dimethylaminoazobenzene (Kline, Miller, Rusch and Baumann, 1946), or polycyclic hydrocarbons (Tannenbaum, 1944). In this latter case it has been shown that the animals on a high fat diet eat more and thus a high calorie intake is achieved leading to a situation similar to that considered in the previous paragraph. Even when allowance is made for this increased calorie intake there is still a slight augmentation. A possible clue to this effect may lie in the finding by

Kaunitz, Johnson and Slanetz (1952) that rancid fat can accelerate the utilisation of riboflavin, an agent known to be protective against certain types of tumour formation and also able to affect –SH levels (see above).

The best known example of an enhancing factor is the use of croton oil in skin painting experiments after a minimal dose of carcinogen. Calcutt and Coates (1961) have shown that after a single application of 7,12-dimethylbenzanthracene to mouse skin there is only a transient rise in the skin –SH level and then a return to the normal level. If this single treatment which would be expected to yield no—or very few—tumours is followed by repeated paintings with croton oil then a sustained rise in –SH levels similar to that achieved by repeated paintings with dimethylbenzanthracene is achieved. Thus, after an initiating treatment two different agents, one a carcinogen and the other a non carcinogen, produce the same biochemical effect and the same biological result.

*Anticarcinogens and sulphydryl groups*

Considerable attention has been given at various times to the search for agents which will inhibit the formation of experimental tumours. In a number of cases specific agents have been used and a clear cut evaluation of results is possible, but in many cases the number of factors involved is such as to make interpretation difficult if not impossible.

Although never substantiated experimentally the idea that carcinogens became linked to tissue sulphydryl groups has long been current. On this basis Crabtree (1944, 1945, 1946) investigated a series of compounds known to inhibit sulphydryl groups or to form mercapturates. These included bromobenzene, maleic and citraconic anhydrides, anthracene and phenanthrene. When painted on to mouse skin concurrently with carcinogenic polycyclic hydrocarbons they reduced the expected tumour yields. It was argued that these compounds reduced the availability of tissue –SH for binding with the carcinogen. In the light of evidence considered earlier it can now be suggested that they limit the rise in tissue –SH level induced by the carcinogen. Further findings which may be considered similarly are those of Carruthers (1940), Riley and Wallace (1941) and Riley and Pettigrew (1944), that aldehydes inhibit carcinogenesis since Schubert (1936) has shown aldehydes to react with thiol compounds. Mustard gas is another compound which reacts with –SH groups and this was found by Berenblum (1935) to inhibit the induction of tar tumours.

On the alternate policy of offering sulphydryl which would compete with that in the tissue for carcinogens Crabtree (1948) tested a series of monothiols and also BAL (2 : 3 dimercapto-propanol) against tumour induction by polycyclic hydrocarbons. The monothiols were found to be ineffective but the BAL did show slight activity. This was confirmation of the experience of Lusky, Braun and Woodard (1947) who had obtained a noticeable reduction in the yield of benzopyrene induced mouse skin tumours by concurrent treatment with BAL. Earlier, Reimann and Hall (1936) claimed that thiocresol effectively inhibited carcinogenesis by 1,2 : 5,6-dibenzanthracene but Crabtree (1948) pointed out that under the experimental conditions used this conclusion was not warranted. In connection with these experiments a further point derives from the fact that Calcutt (1949) and Garzia and Dansi (1953) found that benzopyrene was itself oxidised in the presence of autoxidising thiols. Under these circumstances the application of a thiol to mouse skin already treated with benzopyrene may have resulted in oxidation of both the carcinogen and thiol and a consequent reduction in the effective dose of carcinogen.

A rather different type of anticarcinogen is Vitamin A. This was found by Rosicky and Hatschek (1943) to diminish the yield of mouse skin benzopyrene tumours when painted concurrently with the carcinogen. It has since been claimed by Flesch and Goldstone (1952) that Vitamin A inactivates epidermal –SH groups. Pelc and Fell (1960) have found excess Vitamin A to severely limit the uptake of cystine throughout the epithelium of chick embryo. This might indicate a limitation of substrate available for the formation of –SH groups.

Reference has already been made to the general lowering of tissue glutathione levels brought about by hypophysectomy. This procedure has also been found to profoundly influence the production of experimental tumours. Both delay in the appearance of a tumour and total suppression of tumour formation have been recorded. The findings in these experiments have been summarised in Table III.

TABLE III.—*The Influence of Hypophysectomy upon Experimental Tumour Induction*

| Agent | Animal | Site | Result | Reference |
|---|---|---|---|---|
| 3,4-Benzopyrene . . . . | Mouse | Skin | Reduced yield | Kortweg and Thomas (1939). |
| 3,4-Benzopyrene . . . . | Rats | Subcut. tissue | Delay in appearance | Agate, Antopol, Glaubach, Agate and Graff (1955). |
| 20-Methylcholanthrene . . . | ,, | Muscle | Reduced yield | Moon and Simpson (1955). |
| 9,10-Dimethylbenzanthracene . . | ,, | ,, | ,, ,, | Moon, Li and Simpson (1956). |
| 9,10-Dimethylbenzanthracene . . | ,, | ,, | Delay in appearance | Noble and Walters (1954). |
| 3′-Methyl, 4-dimethylaminoazobenzene | ,, | Liver | Reduced yield | Robertson, O'Neal, Griffin and Richardson (1953). |
| N.2 Fluoroenyldiacetamide . . | ,, | ,, | No tumours* | O'Neal, Hoffman, Dodge and Griffin (1958). |

* High incidence of tumours of Harderian gland origin.

*Human preneoplastic conditions in relation to sulphydryl levels*

Gradually it has become recognised that certain human disease states are associated with a high incidence of tumour occurrence.

A well recognised condition is that of oral leucoplakia, and this has been found by Abels, Rekers, Martin and Rhoads (1942) to be associated with dietary insufficiencies of Vitamins A and $B_2$ (riboflavin) and often to respond to improved diet. Evidence has already been given implicating Vitamin A and B levels with the sulphydryl content of tissues. It is under conditions of low vitamin levels that increased –SH levels might be expected, and in this case there is an association of low vitamin levels with a precancerous state.

A deficiency of riboflavin has also been associated with the Patterson-Kelly (Plummer-Vinson) syndrome by Ahlbom (1936). This condition is believed to predispose to carcinoma of the nasopharynx. Vitamin deficiency has also been incriminated in the aetiology of liver cancer amongst the Bantus (Berman, 1951).

Stocks and Karn (1933) concluded that the regular consumption of milk, green vegetables and possibly wholemeal bread had some kind of protective influence against cancer. It may not be without point that these foodstuffs are major sources of riboflavin.

*Sulphydryl levels at the end of the latent period*

In the earlier part of this paper, evidence was offered that a rise in the target tissue –SH level occurs during the latent period. This rise was often of a duration much shorter than the latent period for the carcinogen and tissue in question. The question as to what happens to –SH levels up to the appearance of the tumour must be considered. The available evidence is limited. Boyland and Mawson (1938) continued their glutathione estimations on the livers of mice treated with 3,4 : 5,6-dibenzcarbazole until bile duct proliferation occurred. After 120 days most values had returned to the normal level. Fiala and Fiala (1959) in their estimations of glutathione in azodye treated rat liver, found that after the rise period (30–80 days) there was a progressive decline up to 210 days after initiation of treatment. Although the experiments were not carried on long enough there are indications in some of the results of Calcutt, Doxey and Coates (1960, 1961) that after the rise period there is a fall to below normal levels.

There are, additionally, some indications that this reduced –SH level persists into the formed tumour. Thus, Fiala and Fiala (1959) found much lower levels of glutathione in both azo dye induced hepatomas and the Novikoff hepatoma as compared with normal rat liver. Kinosita (1938) found primary rat hepatoma to have a lower glutathione content than rat liver. Greenstein and Leuthardt (1945) found the total cysteine content of four different rat tumours to be rather lower than that of normal rat tissues. Doxey (unpublished data) has found azo dye induced rat hepatoma to have both a lowered glutathione and lowered protein –SH content as compared with normal rat liver. Benzopyrene induced epitheliomata of mouse skin also had a low total –SH content as compared with normal mouse skin. Several transplantable mouse tumours were also found to have rather low total –SH values.

*Sulphydryl levels in relation to stages of carcinogenesis*

It is now generally accepted that the carcinogenic process involves at least two different stages. These are usually referred to as " initiation " and " promotion ". The data which has already been collected must also be considered in terms of these stages in the carcinogenic process.

Initiation usually only requires a single treatment with the carcinogenic agent. It was found by Di Paolo and Niedbala (1957) that single applications of 1,2 : 5,6-dibenzanthracene or 7,12-dimethylbenzanthracene caused temporary –SH rises in mouse skin –SH levels. Calcutt and Coates (1961) obtained a similar result with 7,12-dimethylbenzanthracene. Di Paolo and Niedbala (1957) also recorded slight falls in –SH level shortly after application of the hydrocarbons. These may have been associated with metabolism of the hydrocarbons since Calcutt, Doxey and Coates (1959) associated similar slight falls in mouse liver –SH values after treatment with carcinogenic hydrocarbons with detoxication processes. Examination of the figures in Table I suggests that in many cases there has been no effect whatever until several applications of the agent have been made. On the basis of this restricted evidence no conclusions can be drawn as to any immediate effects upon –SH levels. Calcutt and Coates (1961) have pointed out that prior treatment with dimethylbenzanthracene does appear to affect the timing of a later response to croton oil treatment. This however does not necessarily imply an initial effect upon sulphydryl groups themselves.

When after an initial treatment with a carcinogen further applications of the same agent or of croton oil are made there is a well defined elevation of –SH levels. This suggests that the main response is during the promotion phase. Whether the apparent later decline in –SH levels to below normal values should be regarded as part of this process or as an independent stage is uncertain.

The whole picture in terms of sulphydryl levels can be summarised pictorially as in Fig. 1.

FIG. 1.—Changes in tissue sulphydryl levels during carcinogenesis by chemical agents.

GENERAL DISCUSSION

The evidence which has been quoted above offers a reasonable basis for the overall picture of the behaviour of tissue –SH groups during the carcinogenic process. That there is a direct association between the carcinogenic process and –SH levels is attested by the target tissue specificity of the response to carcinogens and the inhibitory effect of known –SH inhibitors upon the carcinogenic response. Whether the actual tissue sulphydryl content is directly involved in carcinogenesis or whether the –SH level is merely symptomatic of some other more essential tissue change is unknown. Certainly it seems very unlikely that –SH levels could be altered without concomitant changes in enzymatic and other cellular activities. The –SH measurements, however, do offer a direct estimate of the biochemical status of the tissue from the time of initiation through to the emergence of the developed tumour, and as such offer considerable scope for the further assessment of factors influencing carcinogenesis.

Evidence for the origin of the –SH groups which appear in tissues during the early stages of treatment with carcinogenic chemicals or cocarcinogens is lacking. Again the question as to what happens to this material during the later stages of carcinogenesis still requires an answer.

This paper has collected together data from many and varied sources. Much of this information is superficially unrelated but on the basis of involvement with sulphydryl levels a unifying factor is apparent. The original hypothesis that an elevated sulphydryl level in the target tissue is an essential for tumorigenesis has been found consistent with other data in the literature. At the same time it is suggested that a more complete picture would be one of elevation of tissue –SH levels followed by a fall to subnormal values and the persistence of this latter value into the formed tumour.

No excuse is offered for this paper being speculative in parts ; it is merely hoped that this will help to stimulate further experimental work and the publication of data which will help to confirm or refute the ideas which have been expressed.

SUMMARY

1. The evidence for chemical carcinogens causing a rise in target tissue sulphydryl levels has been summarised.

2. It has been shown that many agents or factors promoting or inhibiting carcinogenesis can affect tissue –SH levels in a manner which is predictable on the hypothesis that elevated tissue –SH is an essential part of the carcinogenic process.

3. It is concluded that during carcinogenesis a period of elevated tissue –SH occurs and is followed by a period of subnormal levels. This latter subnormal level is persistent into the resultant tumour.

4. Influence upon sulphydryl levels is a unifying factor which connects much apparently unrelated experimental data.

I am indebted to Mr. D. Doxey of this Department, for permission to quote from his umpublished experimental results.

The expenses of this work were defrayed from a block grant from The British Empire Cancer Campaign.

### REFERENCES

ABELS, J. C., REKERS, P. E., MARTIN, H. AND RHOADS, C. P.—(1942) *Cancer Res.*, **2**, 381.

AGATE, F. J., ANTOPOL, W., GLAUBACH, SUSI, AGATE, FAY AND GRAFF, S.—(1955) *Ibid.*, **15**, 6.

AHLBOHM, H. E.—(1936) *Brit. med. J.*, ii, 331.

BAUMANN, C. A.—(1948) *J. Amer. diet. Ass.*, **24**, 573.

BERENBLUM, I.—(1935) *J. Path. Bact.*, **40**, 549.

BERMAN, C.—(1951) ' Primary Carcinoma of the Liver '. London (H. K. Lewis).

BOYLAND, E. AND MAWSON, ELINOR, H.—(1938) *Biochem. J.*, **32**, 1460.

BYERRUM, R. U., ERWAY, H. F. AND DUBOIS, K. P.—(1948) *Arch. Biochem.*, **17**, 41.

CALCUTT, G.—(1949) *Brit. J. Cancer*, **3**, 306.—(1961*a*) *Ibid.*, **15**, 157.—(1961*b*) *Ibid.*, **15**, 157.—(1961*c*) *Naturwissenschaften*, in press.

CALCUTT, G. AND COATES, JOAN.—(1961) *Brit. J. Cancer*, **15**, 360.
*Idem* AND DOXEY, D.—(1959) *Exp. Cell Res.*, **17**, 542.
*Iidem* AND COATES, JOAN.—(1959) *Brit. J. Cancer*, **13**, 711.—(1960) *Ibid.*, **14**, 746.—(1961*a*) *Ibid.*, **15**, 149.—(1961*b*) *Nature*, **192**, 164.
CARRUTHERS, G.—(1940) *Arch. Path.*, **30**, 1184.
CRABTREE, H. G.—(1944) *Cancer Res.*, **4**, 688.—(1945) *Ibid.*, **5**, 346.—(1946) *Ibid.*, **6**, 553.—(1948) *Brit. J. Cancer*, **2**, 281.
DI PAOLO, J. A. AND NIEDBALA, T. F.—(1957) *Proc. Soc. exp. Biol. N.Y.*, **96**, 255.
FIALA, S. AND FIALA, ANNA E.—(1959) *Brit. J. Cancer*, **13**, 136.
FLESCH, P. AND GOLDSTONE, S. B.—(1952) *J. invest. Derm.*, **18**, 267.
GALLICO, E. AND BORETTI, G.—(1948) *Tumori*, **34**, 130.
GARZIA, A. AND DANSI, A.—(1953) *Il Pharmaco*, **8**, 449.
GOSS, H. AND GREGORY, P. W.—(1935) *Proc. Soc. exp. Biol. N.Y.*, **32**, 681.
GREENSTEIN, J. P. AND LEUTHARDT, F. M.—(1945) *J. nat. Cancer Inst.*, **5**, 111.
GREGORY, P. W. AND GOSS, H.—(1934) *J. exp. Zool.*, **69**, 13.
IKI, H.—(1939) *Gann*, **33**, 216, quoted by COOK, J. W. AND KENNAWAY, E. L.—(1940) *Amer. J. Cancer*, **39**, 381.
KAUNITZ, H., JOHNSON, R. E. AND SLANETZ, C. A.—(1952) *J. Nutr.*, **46**, 151.
KINOSITA, R.—(1938) *J. Japan Gastroenterol. Soc.*, **37**, 513, quoted by GREENSTEIN J. P.—(1954) ' Biochemistry of Cancer '. New York (Academic Press).
KLINE, B. E., MILLER, J. A., RUSCH, H. P. AND BAUMANN, C. A.—(1946) *Cancer Res.*, **6**, 5.
KORTWEG, R. AND THOMAS, F.—(1939) *Amer. J. Cancer*, **37**, 36.
LAZAROW, A.—(1954) in ' Glutathione, a Symposium '. New York (Academic Press).
LUSKY, L. M., BRAUN, H. A. AND WOODARD, G.—(1947) *Cancer Res.*, **7**, 667.
MASAYAMA, I., IKI, H., YOKAYAMA, T. AND HASIMOTO, M.—(1938) *Gann*, **32**, 303, quoted by COOK, J. W. AND KENNAWAY, E. L.—(1940) *Amer. J. Cancer*, **39**, 381.
MOON, H. D., LI, C. H. AND SIMPSON, MIRIAM, E.—(1956) *Cancer Res.*, **16**, 111.
*Idem* AND SIMPSON, MIRIAM, E.—(1955) *Ibid.*, **15**, 403.
NOBLE, R. L. AND WALTERS, J. H.—(1954) *Proc. Amer. Ass. Cancer Res.*, **1**, 35.
O'NEAL, M. A., HOFFMANN, H. E., DODGE, B. G. AND GRIFFIN, A. C.—(1958) *J. nat. Cancer Inst.*, **21**, 1161.
PELC, S. R. AND FELL, HONOR, B.—(1960) *Exp. Cell Res.*, **19**, 99.
REIMANN, S. P. AND HALL, E. M.—(1936) *Arch. Path.*, **22**, 55.
RILEY, J. F. AND PETTIGREW, F.—(1944) *Cancer Res.*, **4**, 502.
*Idem* AND WALLACE, A. B.—(1941) *Brit. J. exp. Path.*, **22**, 24.
ROBERTSON, C. H., O'NEAL, M. A., GRIFFIN, A. C. AND RICHARDSON, H. L.—(1953) *Cancer Res.*, **13**, 776.
RONDONI, P. AND BORETTI, G.—(1947) *Tumori*, **33**, 274.
ROSICKY, J. AND HATSCHEK, R.—(1943) *Z. Krebsforsch.*, **54**, 26.
SCHUBERT, M. P.—(1936) *J. biol. Chem.*, **114**, 341.
SCHULZE, G.—(1960) *Z. ges. exp. Med.*, **133**, 194.
SETÄLÄ, K., ÄYRÄPÄÄ, O., NISKANEN, E. E., STJERNVALL, L. AND NYHOLM, M.—(1960) *Naturwissenschaften*, **47**, 357.
STOCKS, P. AND KARN, MARY N.—(1933) *Ann. Eugen., Lond.*, **5**, 237.
TANNENBAUM, A.—(1944) *Cancer Res.*, **4**, 683.
*Idem* AND SILVERSTONE, H.—(1957) Chapter 9 in ' Cancer '. Edited by Raven. London (R. W. Butterworth).
WOOD, J. L. AND KRAYNAK, M. E.—(1953) *Cancer Res.*, **13**, 358.